# Upcycled Cocoa Pod Husk: A Sustainable Source of Phenol and Polyphenol Ingredients for Skin Hydration, Whitening, and Anti-Aging

**DOI:** 10.3390/life15071126

**Published:** 2025-07-17

**Authors:** Aknarin Anatachodwanit, Setinee Chanpirom, Thapakorn Tree-Udom, Sunsiri Kitthaweesinpoon, Sudarat Jiamphun, Ongon Aryuwat, Cholpisut Tantapakul, Maria Pilar Vinardell, Tawanun Sripisut

**Affiliations:** 1 Cosmetics for Beauty and Wellness Research Unit, Mae Fah Luang University, Chiang Rai 57100, Thailand; p.aknarin@gmail.com (A.A.); setinee.cha@mfu.ac.th (S.C.); thapakorn.tre@mfu.ac.th (T.T.-U.); 2 School of Cosmetic Science, Mae Fah Luang University, Chiang Rai 57100, Thailand; sunsiri.kitthaweesinpoon@gmail.com (S.K.); sudarat.jia@mfu.ac.th (S.J.); ongon.bl1092@gmail.com (O.A.); 3 School of Science, Walailak University, Nakhon Si Thammarat 80160, Thailand; cholpisut.ta@mail.wu.ac.th; 4 Departament de Bioquimica i Fisiologia, Facultat de Farmacia i Ciències de l’Alimentació, Universitat de Barcelona, 08028 Barcelona, Spain

**Keywords:** cocoa pod husk, *Theobroma cacao* L., phenol, hydration, whitening, anti-aging

## Abstract

*Theobroma cacao* L. (cocoa) pod husk, a byproduct of the chocolate industry, has potential for commercial applications due to its bioactive compounds. This study aimed to determine the phytochemical composition, biological activity, and clinical efficacy of a standardized extract. This study compared 80% ethanol (CE) and 80% ethanol acidified (CEA) as extraction solvents. The result indicated that CEA yielded higher total phenolic content (170.98 ± 7.41 mg GAE/g extract) and total flavonoid content (3.91 ± 0.27 mg QE/g extract) than CE. Liquid chromatography–tandem mass spectrometry (LC/MS/MS) identified various phenolic and flavonoid compounds. CEA demonstrated stronger anti-oxidant (IC_50_ = 5.83 ± 0.11 μg/mL in the DPPH assay and 234.17 ± 4.01 mg AAE/g extract in the FRAP assay) compared to CE. Additionally, CEA exhibited anti-tyrosinase (IC_50_ = 9.51 ± 0.01 mg/mL), anti-glycation (IC_50_ = 62.32 ± 0.18 µg/mL), and anti-collagenase (IC_50_ = 0.43 ± 0.01 mg/mL), nitric oxide (NO) production inhibitory (IC_50_ = 62.68 μg/mL) activities, without causing toxicity to cells. A formulated lotion containing CEA (0.01–1.0% *w*/*w*) demonstrated stability over six heating–cooling cycles. A clinical study with 30 volunteers showed no skin irritation. The 1.0% *w*/*w* formulation (F4) improved skin hydration (+52.48%), reduced transepidermal water loss (−7.73%), and decreased melanin index (−9.10%) after 4 weeks of application. These findings suggest cocoa pod husk extract as a promising active ingredient for skin hydrating and lightening formulation. Nevertheless, further long-term studies are necessary to evaluate its efficacy in anti-aging treatments.

## 1. Introduction

Cocoa (*Theobroma cacao* L.) is a globally significant crop, primarily cultivated for chocolate production [1]. In 2021, global cocoa production reached approximately 5.024 million tons, with the industry projected to grow by 6.3% from 2020 [2]. The global market size of cocoa products is expected to expand at a compound annual growth rate (CAGR) of 1.7% in the forecast period of 2022 to 2025 and is expected to reach USD 22.59 billion by 2025, up from USD 21.13 billion in 2019 [3]. As of 2025, the global chocolate market is valued at approximately USD 114 billion and is projected to reach USD 145 billion by 2030, reflecting a compound annual growth rate (CAGR) of 4.95%. Emerging market trends indicate a growing consumer preference for high-quality, functional chocolate products, with increasing emphasis on premium, sustainable, and health-oriented formulations [4]. In Thailand, the majority of cocoa plantations are located in the northern region, making up 43% of the country’s total plantation area. Chiang Rai, Chiang Mai, and Phitsanulok Provinces have the highest levels of cocoa cultivation. During cocoa processing, a large volume of biomass, particularly cocoa pod husk, which constitutes about 70–75% of the dry weight of the fruit. This byproduct is typically discarded on plantations, posing environmental concerns and increasing the risk of disease outbreaks such as black pod rot [5,6]. However, cocoa pod husk can be converted into a value-added material because they are rich in bioactive compounds. Previous studies have demonstrated that cocoa pod husk contains high levels of phenols and polyphenols such as protocatechuic acid, p-hydroxybenzoic acid, salicylic acid, kaempferol, and resveratrol, which exhibit antioxidant and tyrosinase inhibitory activities [7,8].

The utilization of cocoa pod husk as a source of functional ingredients aligns with the growing interest in sustainable practices and the value-added conversion of agricultural waste. This approach not only supports the development of eco-friendly cosmetic and pharmaceutical applications but also contributes to waste reduction and promotes circular economy initiatives in cocoa-producing regions such as Thailand [9,10,11]. Although interest in the valorization of cocoa byproducts has expanded significantly in recent years, there is still a lack of comprehensive investigations comparing ethanol-based extraction solvents, particularly acidified ethanol, with respect to their efficiency in isolating bioactive compounds relevant to cosmetic use [12,13]. Conventional extraction methods, such as aqueous and ethanol extraction, have been widely applied. However, the systematic optimization of solvent systems to maximize bioactive yield remains insufficiently explored. Moreover, few studies have correlated extract bioactivity with clinical cosmetic performance [14,15]. This study aimed to compare the efficacy of 80% ethanol and acidified ethanol (pH 3) as extraction solvents for cocoa pod husk, with a specific focus on evaluating their efficiency in yielding bioactive chemical constituents. The investigation assessed multiple cosmetic-related bioactivities, including antioxidant capacity, anti-glycation, anti-tyrosinase, anti-collagenase, and suppression of nitric oxide (NO) production. Additionally, clinical evaluations were conducted to determine the extract’s potential efficacy in enhancing skin hydration, whitening, and anti-aging effects when incorporated into cosmetic formulations. This research provides novel insights into sustainable cosmetic ingredient development using Thai cocoa byproducts.

## 2. Materials and Methods

### 2.1. Materials and Reagents

All chemicals and reagents used in this study were of analytical grade. Deionized water from Scientific & Technological Instruments Centers, MFU (Chiang Rai, Thailand), and 95% ethanol were purchased from Northern Chemicals and Glasswares Ltd., Part. (Chiang Mai, Thailand). Acetic acid, sodium acetate, sodium carbonate, concentrated hydrochloric acid (HCl), ferric chloride, formic acid, acetonitrile, and methanol were purchased from RCI Labscab (Bangkok, Thailand). Folin–Ciocalteu’s reagent was purchased from LOBA Chemie (Mumbai, India). Quercetin, kojic acid, gallic acid, L-(+)-ascorbic acid, mushroom tyrosinase, L-DOPA, 2,2-diphenyl-1-picrylhydrazyl (DPPH), and 2,4,6-tri(2-pyridyl)-1,3,5-triazine (TPTZ) were purchased from Sigma-Aldrich, St. Louis, MO, USA. Additional reagents included aluminium chloride (AlCl_3_), disodium hydrogen phosphate (Na_2_HPO_4_), and sodium dihydrogen phosphate (NaH_2_PO_4_) from QReC Co., Ltd., Auckland New Zealand; disodium EDTA and triethanolamine from Chemipan Corporation Co., Ltd., Bangkok, Thailand and acrylates/C10-30 alkyl acrylate crosspolymer (Carbopol Ultrez-21) from Lubrizol, Singapore. Glycerin was obtained from Thai Glycerin Co., Ltd., Samut Sakhon, Thailand. Propylene glycol from Shell Chemicals, Deer Park, TX, USA, and PEG-40 hydrogenated castor oil from Nikko Chemicals Co., Ltd., Singapore. Dimethicone was purchased from Dow Chemicals Thailand Ltd., Rayong, Thailand, and caprylic/capric triglyceride from INOLEX Incorporated, Philadelphia, PA, USA. Isononyl isononanoate, Bis-PEG/PPG-16/16 PEG/PPG-16/16 dimethicone (Silk Lotion Maker™), and phenoxyethanol (and) ethylhexylglycerin were supplied by Chanjao Longevity Co., Ltd., Bangkok, Thailand. Viscolam AT 100 P (sodium polyacryloyldimethyl taurate, hydrogenated polydecene, and trideceth-10) was obtained from Krungthepchemi Co., Ltd., Bangkok, Thailand.

### 2.2. Plant Material Collection and Preparation

**Figure 1:**
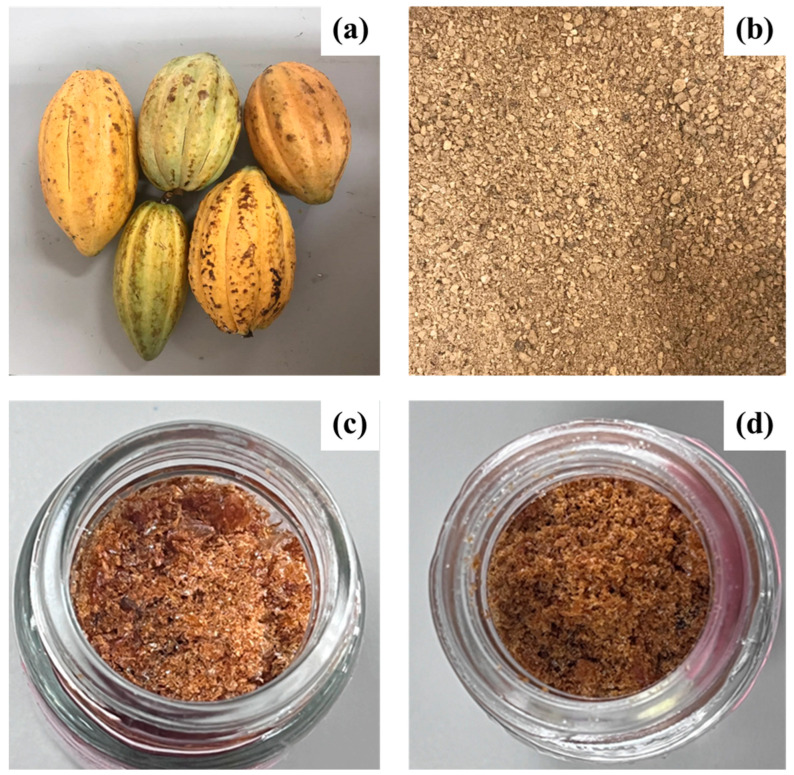
Sample preparation and cocoa pod husk (CPH) extracts. (**a**) Fresh cocoa fruits, (**b**) dried and ground cocoa pod husk powder, (**c**) CPH extract obtained from CE, (**d**) CPH extract obtained using CEA.

Fresh cocoa (Chumporn Hybrid 1: Pa7 × Na32) fruits (*Theobroma cacao* L.) were collected from Fill for Garden House Cocoa Farm, a local farm in Mae Sai District, Chiang Rai, Thailand (Latitude 20.3011428, Longitude 99.9148956). The cocoa trees used in this study were four years old, and the fruits were harvested at four months of maturity. Fruits were selected based on uniform shape, size, and color, as shown in Figure 1a. After harvesting, the fruits were thoroughly washed with tap water, and only the cocoa pod husks were separated for use. The husks were chopped into small pieces and dehydrated in a tray dryer at 60 °C for 24 h. The dried husks were then ground into a fine powder using an electric blender (EM-ICE, Sharp, Sakai, Japan). The appearance of cocoa pod husk (CPH) powder is shown in Figure 1b.

### 2.3. Cocoa Pod Husk Extract Preparation

The CPH powder was extracted by maceration using 80% ethanol (CE) and 80% ethanol acidified (CEA) as solvents. The extraction method was modified from [5]. A total of 2.5 g of CPH powder was added to 500 mL of each solvent and shaken at 120 rpm at 40 °C in an incubator shaker for 30 min. After filtration with Whatman paper No.1, the filtrate was evaporated to remove the solvent using a rotary evaporator. The extract was then lyophilized by freeze-dryer and stored at −20 °C until further use.

### 2.4. Total Phenolic Content

Total phenolic content (TPC) was measured using the Folin–Ciocalteu spectrophotometric method, as described by [16], with slight modification. Briefly, 20 μL of the sample or standard was added to a 96-well plate, followed by 100 μL of Folin–Ciocalteu’s reagent. The mixture was allowed to stand for 5 min. Then, 80 μL of 7.5% sodium carbonate solution was added, and plate was kept in the dark at room temperature for 30 min. Absorbance was measured at 765 nm using a microplate reader. A standard calibration curve was obtained from gallic acid in the concentration range of 0.01–0.5 mg/mL. TPC results were expressed as mg gallic acid equivalents (GAEs) per gram of extract (GAE/g extract).

### 2.5. Total Flavonoid Content

Total flavonoid content (TFC) of cocoa pod husk extracts was determined using the aluminium chloride colorimetric method as described by [16], with slight modifications. Briefly, 50 μL of sample or standard was added to a 96-well plate, followed by 130 μL of 80% ethanol, 10 μL of 10% aluminium chloride, and 10 μL of 1M sodium acetate. The mixture was incubated in the dark at room temperature for 40 min. Absorbance was measured at 415 nm using a microplate reader. Quercetin was used as a standard for the calibration curve. TFC results were expressed as mg of quercetin equivalents (QEs) per gram of extract (mg QE/g extract).

### 2.6. LC-MS/MS Analysis

LC-MS/MS analysis was used to qualitatively analyze the chemical composition of cocoa pod husk. It is equipped with a reversed-phase C_18_ analytical column of 2.1 mm × 50 mm × 1.7 μm particle size. The column oven temperature was set at 30 °C. Eluent A was a solution of 0.1% formic acid (*v*/*v*) in water. Eluent B was a solution of 0.1% formic acid (*v*/*v*) in acetonitrile. The gradient profile was set as follows: 0–1 min, 95%A/5%B; 1–6 min, 83%A/17%B; 6–35 min, 100%B; 35–45 min, 95%A/5%B. The injection volume was 2 μL with a flow rate of 0.2 mL/minute, and the run time was 45 min. The UHPLC was hyphenated to mass spectrometer with an electrospray ionization interface set at positive mode. Nitrogen is used as a collision and spray gas.

### 2.7. Antioxidant Activities

#### 2.7.1. DPPH Free-Radical Scavenging Assay

The antioxidant activity was evaluated using the DPPH free-radical scavenging assay, adapted for a 96-well plate, and modified from [17]. Briefly, 100 μL of sample or standard was added to each well of a 96-well plate, followed by 100 μL of DPPH solution (0.06 mM). The mixture was incubated in a dark place at room temperature for 30 min. Absorbance was then measured at 517 nm using a microplate reader. Ascorbic acid was used as a positive control. The DPPH scavenging activity was expressed as the percentage of DPPH radical inhibition and half maximal inhibitory concentration (IC_50_).

#### 2.7.2. Ferric Acid Reducing Power

Ferric-Reducing Antioxidant Power (FRAP) assay of the extracts was determined using a modified method based on [18]. The FRAP reagent was prepared by mixing the following three different solutions: 10 mmol/L 2,4,6-tri (2- pyridyl)-1,3,5-triazine (TPTZ), 300 mmol/L acetate buffer of pH 3.6, and 20 mmol/L FeCl3 at 1:10:1 ratio. The assay was done by mixing 50 μL of diluted extracts with 200 μL of the FRAP reagent. The mixture was then incubated at 37 °C for 5 min, and absorbance was measured at 620 nm using a microplate spectrophotometer. L-(+)-ascorbic acid was used as a standard. Antioxidant activity by FRAP assay was expressed as mg of ascorbic acid equivalents (AAEs) per gram of extract (mg AAE/g extract).

### 2.8. Anti-Glycation Activity

Anti-glycation activity was evaluated using the method described by [19,20]. The diluted extract solution, BSA (10 mg/mL), and D-fructose (90 mg/mL) were each prepared separately in 50 mM phosphate buffer (pH 7.4). Then, 1 mL of each solution was mixed and incubated in the dark at 37 °C for 72 h. Fluorescence intensity was measured at excitation and emission wavelengths of 360 and 420 nm using an Infinite M nano microplate reader (Tecan, Männedorf, Zürich, Switzerland). Quercetin was used as a positive control. Anti-glycation activity was expressed as the percentage of inhibition and half maximal inhibitory concentration (IC_50_).

### 2.9. Tyrosinase Inhibitory Activity

In vitro skin whitening effect of extracts was evaluated based on mushroom tyrosinase inhibition using L-DOPA as a substrate, using the method as described by [21], with modifications. Firstly, the sample was diluted in phosphate buffer solution (50 mM, pH 6.8). Afterwards, 1650 μL of phosphate buffer solution was added into a test tube, followed by 300 μL of mushroom tyrosinase solution (480 U/mL in PBS pH 6.8) and 300 μL of sample. After incubation for 10 min at room temperature, 750 μL of L-DOPA (0.85 mM in PBS pH 6.8) was added. The mixture was further incubated for 20 min. After that, the mixture was immediately transferred to a cuvette, and the absorbance was measured at 490 nm using a UV-Vis spectrophotometer. Kojic acid was used as a reference standard. The concentration of standard or sample that can inhibit the tyrosinase activity by 50% (IC_50_) was calculated from the graph plotted between the percentage of tyrosinase inhibition and concentration of standard or sample.

### 2.10. Anti-Collagenase Activity

The collagenase inhibitory potential of cocoa pod husk extracts was evaluated using an enzyme-substrate assay, adapted from the method described by [22]. For the enzymatic reaction, a 50 mM Tricine buffer (pH 7.5) containing 400 mM NaCl and 10 mM CaCl_2_ was used as the solvent. Briefly, a mixture of 0.5 units/mL collagenase and the sample solution was prepared and allowed to incubate for 15 min. Following incubation, 2.0 M FALGPA was added, and the absorbance at 340 nm was immediately recorded in kinetic mode for 20 min using a UV-Vis spectrophotometer. Epigallocatechin gallate (EGCG) was used as a reference standard. Anti-collagenase activity was expressed as the percentage of inhibition and half maximal inhibitory concentration (IC_50_).

### 2.11. Cytotoxicity Using MTT Assay

The cytotoxic potential of the extract was assessed using RAW 264.7 macrophage cells as previously described [23]. Macrophage cells were obtained from the China Center for Type Culture Collection. Briefly, the cells were incubated overnight at 37 °C with 5% CO_2_. Subsequently, the cells were exposed to various concentrations of the extracts. After a 24-h treatment, the medium was then removed, and MTT reagent (5 mg/mL) was added to each well. Plates were incubated for an additional 4 h. The cell viability was measured at 570 nm using a Biochrom EZ Read 400 ELISA microplate reader (Biochrom Ltd., Cambridge, UK). The IC_50_ value was calculated as the concentration required to inhibit 50% of cell viability.

### 2.12. Nitric Oxide (NO) Production Inhibition Activity

The assay was conducted following a previously established protocol [23]. RAW 264.7 macrophage cells were plated at a density of 4 × 10^4^ cells per well in 96-well plates and incubated overnight at 37 °C with 5% CO_2_. The next day, cell inflammation was induced with 1 µg/mL lipopolysaccharide (LPS) for 1 h, followed by treatment with diluted concentrations of extracts for 24 h. After incubation, 100 µL of Griess reagent was added to sample and incubated for 10 min. Nitric oxide levels were determined by measuring absorbance at 570 nm using a Biochrom EZ Read 400 ELISA microplate reader (Biochrom Ltd., Cambridge, UK). The half-maximal inhibitory concentration (IC_50_) values were calculated.

### 2.13. Formulation and Stability Evaluation

Standard oil-in-water base lotion without CEA (F1) and lotion formulations containing CEA (F2, F3, F4) were developed. The complete composition of each formulation is presented in Table 1. Briefly, aqueous phase (phase A) and oil phase (phase B) were heated to 75 °C. After that, phase B was added to phase A with continuous stirring. After the emulsion was cooled down to 40 °C, phase C was added and followed by phases D and E. Then, the homogenizer was used to ensure complete homogenization of emulsion.

### 2.14. Clinical Evaluation

#### 2.14.1. Ethical Conduction

The study was conducted in compliance with international guidelines, including Declaration of Helsinki, the Belmont Report, CIOMS Guidelines, and the International Conference on Harmonization of Technical Requirements for Registration of Pharmaceuticals for Human Use—Good Clinical Practice (ICH-GCP). The study protocol was approved by the Mae Fah Luang University Ethics Committee on Human Research with protocol no. EC 22103-17.

#### 2.14.2. Volunteer Recruitment

The study involved thirty healthy male and female volunteers, aged 18–45 years old, with Fitzpatrick skin phototypes 2–5 were recruited for this study. Participants had no history of skin diseases, visible injuries, or anatomical features (such as scars or tattoos) on the test site (left inner forearm) that could interfere with measurements. Subject selection adhered to predefined inclusion criteria, while exclusion criteria comprised pregnancy, lactation, known allergies to test substances, and concurrent participation in other clinical studies. All experimental procedures were comprehensively explained to participants both in written and verbal formats, and informed consent documents were obtained from all subjects prior to study commencement.

#### 2.14.3. Skin Irritation Test

Safety assessment of the formulated lotion containing CEA was evaluated via primary skin irritation assessment using a single application of closed patch test using Finn chamber (8 mm, SmartPractice, Phoenix, AZ, USA). Before applying a patch, the tested area (left inner upper arm) of each volunteer was cleansed with water, dried gently, and allowed to equilibrate for a 30-min period. The six tested substances, including deionized water (negative control), 0.5% *w*/*w* of sodium lauryl sulfate (SLS) solution (positive control), placebo lotion (base lotion without cocoa pod husk extract; F1), lotion formulation containing CEA at three concentration levels, 0.01% *w*/*w* (F2), 0.1% *w*/*w* (F3), and 1.0% *w*/*w* (F4), were applied as a single application under occlusion for a 24 h period. Cutaneous responses were observed and evaluated following patch removal at 30 min, 24 h, 48 h, and 72 h post-exposure. Subsequently, the acute mean irritation index (M.I.I.) was calculated [16].

#### 2.14.4. Clinical Skin Efficacy Evaluation

Twenty-nine healthy volunteers who demonstrated no adverse skin reactions during the skin irritation assessment were enrolled in this clinical efficacy study. All participants were instructed to abstain from applying any cosmetic products to the tested area (left inner forearm), with the sole exception of cleansing products such as soap or body wash, for one week before starting the study, and throughout the complete duration of the study [24]. Each volunteer received four pump bottles of tested lotions, including F1–F4. Participants applied 1 pump of each tested lotion to each designated area of 9 cm^2^ (3 × 3 cm) on their left inner forearm twice daily (morning and evening) for a 28-day (4 weeks) treatment period. Prior to instrumental measurements, subjects were acclimated in an environmentally controlled chamber maintained at (21 ± 2 °C with relative humidity of 55 ± 5% for a minimum equilibration period of 30 min to ensure standardization of cutaneous parameters. Skin hydration was evaluated by Corneometer^®^ CM 825 (Courage + Khazaka, Germany), transepidermal water loss (TEWL) by Tewameter^®^ TM 300 (Courage + Khazaka, Germany), and melanin index by Mexameter^®^ MX 18 (Courage + Khazaka, Germany). Furthermore, the skin surface analysis was conducted by Visioscan^®^ VC98 (Courage + Khazaka, Germany), which performed four surface evaluations of living skin (SELS) parameters: skin smoothness (SEsm), skin roughness (SEr), scaliness (SEsc), and wrinkle (SEw). The skin measurements were performed before the application of tested lotions (baseline; T0) and after the application of tested lotions for 1 (T1), 2 (T2), 3 (T3), and 4 weeks (T4). All the measurements were performed in triplicate.

### 2.15. Statistical Analysis

All data were expressed as mean ± standard deviation (SD), except for clinical efficacy results, which were presented as mean ± standard error of mean (SEM). Statistical analyses of in vitro data were conducted using independent *t*-tests and one-way analysis of variance (ANOVA), followed by Duncan’s New Multiple Range Test (DMRT). Clinical efficacy data were evaluated using paired *t*-tests, Wilcoxon signed-rank test, ANOVA with the Kruskal–Wallis test with Bonferroni correction where appropriate. Differences were considered statistically significant at *p* < 0.05.

## 3. Results and Discussion

### 3.1. Yield

**Figure 2:**
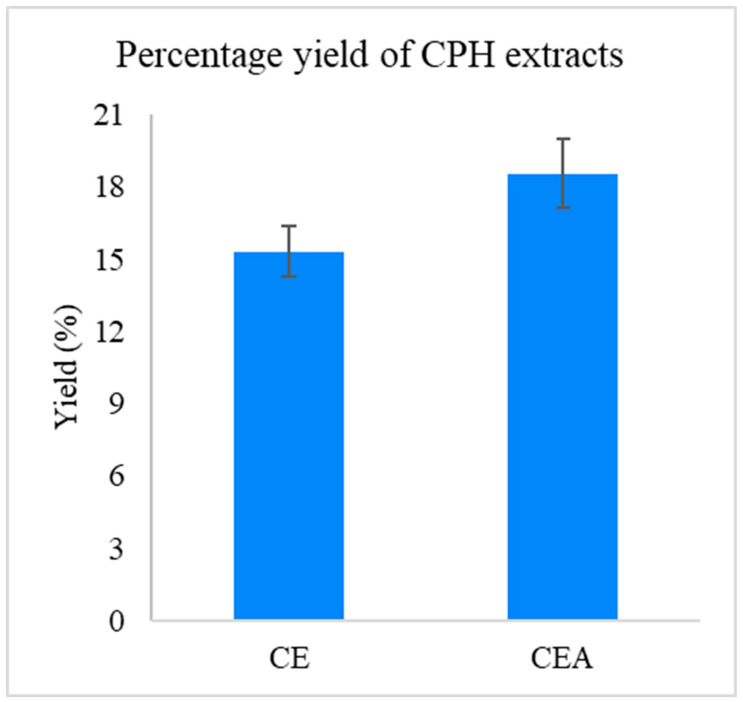
Percentage yield of CPH extracts obtained using 80% ethanol (CE) and 80% ethanol acidified (CEA).

The CPH extracts obtained were brown and solid (Figure 1c,d). The extraction yields using different solvents are shown in Figure 2. The results revealed that CEA provided a significantly higher yield (18.57 ± 1.42%) compared to CE (15.33 ± 1.04%) (*p* < 0.001). These findings align with previous studies that reported higher yields when using acidified solvents [21,25]. Acidification may enhance phenolic compound stability and solubility by promoting cell wall hydrolysis, thereby improving the release and diffusion of these compounds [26].

### 3.2. Total Phenolic Content

After the extractions, the total phenolic content (TPC) of cocoa pod husk extracts was determined and compared among different methods. As shown in Table 2, maceration with CEA produced the highest phenolic content. The acidified solvent enhanced the release of phenolic compounds, which are typically bound to cell wall components. Acidic conditions promote hydrolysis, breaking down plant cell walls and facilitating the solubilization and diffusion of phenolics [26]. This is consistent with [27], who reported increased phenolic extraction under acidified conditions.

### 3.3. Total Flavonoid Content

The total flavonoid content (TFC) of cocoa pod husk extracts is shown in Table 2. Consistent with the TPC results, CEA showed significantly higher TFC than that of CE. Acidic conditions enhance solubility and break down cell matrix components, improving extraction efficiency. According to [5], the cocoa pod husk extract contained various phenolic compounds, including phenolic acids (ferulic acid, chlorogenic acid, protocatechuic acid, etc.), flavonoids (naringenin, kaempferol, apigenin glycosides, procyanidins), and stilbenoids (resveratrol). Acidification of the solvent thus enhances both extraction yield and flavonoid concentration.

### 3.4. Identification of Chemical Composition of CPH Extracts by Using LC-MS/MS

**Figure 3:**
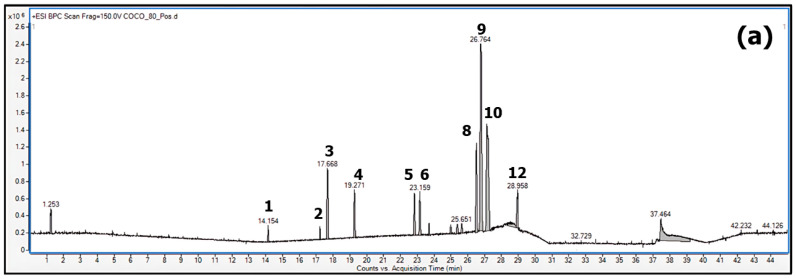

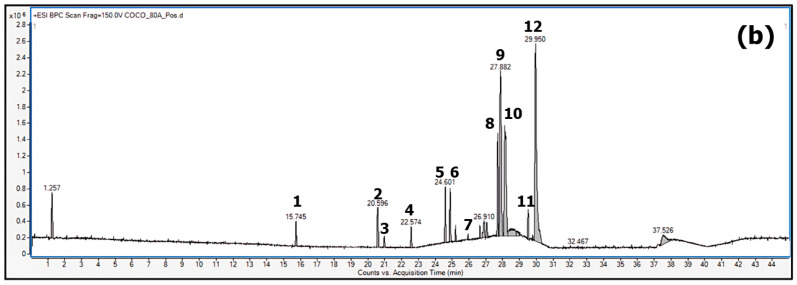
Chromatographic profiles obtained in product ion (positive mode) for CPH extracted by (**a**) 80% ethanol (CE), (**b**) 80% ethanol acidified (CEA), (1) Ferulic acid, (2) Chlorogenic acid, (3) Naringenin, (4) Apig-enin-7-O-glucoside, (5) Procyanidin B1, (6) Procyanidin B2, (7) 2-O-caffeoltartaric acid, (8) Protocatechuic acid, (9) N-[3’,4’-dihydroxy-(E)cinnamoyl]-L-aspartic acid, (10) N-[4’-dihydroxy-(E)cin-namoyl]-L-aspartic acid, (11) Kaempferol, (12) Procyanidin C1.

In the study, metabolites were characterized using an LC-Q-TOF instrument in combination with the METLIN metabolomics spectral library and database. The chemical compositions of cocoa pod husk crudes were analyzed by using the LC-MS/MS technique in positive mode. Each sample was identified based on the literature review in established spectrum databases [28,29,30,31]. All of the crude extracts were analyzed under the same conditions as mentioned above. The chemical composition is listed in Table 3. All crude extracts contained almost similar compounds, including phenolic acid (ferulic acid and procatechuic acid), flavonoid (Narigenin, Apigenin-7-O-glucoside, Procycanidin B1, Procyanidin B2, and Procycanidin C1), and aspartic acid (N-[3′,4′-dihydroxy-(E) cinnamoyl]-L-aspartic acid and N-[4′-dihydroxy-(E) cinnamoyl]-L-aspartic acid) (Figure 3). Therefore, polyphenol compounds were confirmed to be found in all samples. Quantitative analysis was appropriate for further investigation. Moreover, flavonoids are utilized for several cosmetic applications due to their potential antioxidation, anti-inflammatory, anti-mutagenic, and anti-carcinogenic [32].

**Table 3:** Production of cocoa pod husk in positive mode.

Compound	Peak No.	Chemical Formula	RT(min)	Major MS-MS Fragments	a	b	References
Ferulic acid	1	C_10_H_10_O_4_	14.14	176, 152, 144, 116, 105			[28,30]
			14.15		Yes		
			15.74			Yes	
Chlorogenic acid	2	C_16_H_18_O_9_	17.20	337, 181, 163			[28,30]
			17.22		Yes		
			20.59			Yes	
Naringenin	3	C_15_H_12_O_5_	17.66	231, 179, 152, 147			[28,30]
			17.67		Yes		
			20.97			Yes	
Apigenin-7-O-glucoside	4	C_21_H_22_O_10_	19.26	409, 367, 323, 247			[28,30]
			19.97		Yes		
			22.57			Yes	
Procyanidin B1	5	C_30_H_26_O_12_	22.83	407, 339, 289, 248	Yes		[28,30]
			24.60			Yes	
Procyanidin B2	6	C_30_H_26_O_12_	23.14	425, 407, 289, 161, 125			[28,29,31]
			23.15		Yes		
			24.89			Yes	
2-O-caffeoltartaric acid	7	C_13_H_12_O_9_	23.70	312, 296,252			[28]
			25.20			Yes	
Protocatechuic acid	8	C_7_H_6_O_4_	26.51	137, 109, 108	Yes		[28,30]
			26.52				
			27.70			Yes	
N-[3’,4’-dihydroxy-(E)cinnamoyl]-L-aspartic acid	9	C_13_H_13_NO_6_	26.76	163	Yes		[28]
			26.79				
			26.80				
			27.88			Yes	
N-[4’-dihydroxy-(E)cinnamoyl]-L-aspartic acid	10	C_13_H_13_NO_6_	27.14	178, 163	Yes		[28]
			28.13			Yes	
Kaempferol	11	C_15_H_10_O_6_	29.50	257, 229, 149		Yes	[28,30]
Procyanidin C1	12	C_45_H_38_O_18_	28.96	712, 695, 575, 425, 407, 287, 125	Yes		[28,30]
			29.95			Yes	

Crude cocoa pod husk extract (a) 80% ethanol (CE), (b) 80% ethanol acidified (CEA).

### 3.5. Antioxidant Activities

The antioxidant activity of the extracts was assessed using FRAP and DPPH assays. The FRAP assay measures antioxidant potential through the reduction of Fe^3+^-TPTZ to Fe^2+^-TPTZ, resulting in a green-blue color proportional to reducing power [33]. In the DPPH^•^assay, the stable free radical DPPH^•^ changes from dark purple to yellow when reduced by antioxidants [34]. From the result, CEA showed the highest antioxidant capacity, demonstrated by its strongest reducing power and lowest IC_50_ value in the DPPH^•^ assay (Table 4).

### 3.6. Anti-Glycation Activity

The anti-glycation potential was evaluated using a modified Bovine Serum Albumin (BSA) assay, which measures the ability to inhibit the formation of advanced glycation end products (AGEs). AGEs are formed through non-enzymatic reactions between the carbonyl groups of reducing sugars and amino groups of biological macromolecules, and their accumulation is linked to skin damage and accelerated aging [35]. In this study, CEA showed moderate inhibition of AGE formation, with IC_50_ values of 66.96 ± 0.57 µg/mL (Table 4).

### 3.7. Tyrosinase Inhibitory Activity

Tyrosinase inhibition indicates skin-whitening potential, as it reduces melanin production. As shown in Table 4, CEA showed notable tyrosinase inhibitory activity, with an IC_50_ value of 9.51 ± 0.01 mg/mL; however, its efficacy was lower compared to that of kojic acid. Its effect may be due to higher flavonoid content in the acidified extract. Cocoa pod husk contains polyphenols such as ferulic acid, kaempferol, procyanidins, and resveratrol, which contribute to antioxidant and anti-tyrosinase effects [5,36,37]. Tyrosinase inhibition likely occurs via copper ion chelation at the enzyme’s active site, altering its structure [38]. Prior studies reported stronger activity, with IC_50_ values of 357.95 µg/mL [5] and 199.98 µg/mL [8], suggesting that variations in solvents and extraction conditions may influence efficacy.

### 3.8. Anti-Collagenase Activity

Anti-collagenase activity is commonly used as an in vitro assay for evaluating anti-aging potential. A previous study by [5,7] demonstrated that cocoa pod husk extract possessed anti-collagenase activity, attributed to the presence of phenolic compounds and flavonoids. As shown in Table 4, the CEA exhibited anti-collagenase activity with an IC_50_ value of 0.43 ± 0.01 mg/mL, although this was lower than that of the standard compound (EGCG). Interestingly, the anti-collagenase activity of CEA was 3.88 times stronger than that reported by [5].

### 3.9. Cytotoxicity and Nitric Oxide (NO) Production Inhibition Activity

CEA was evaluated for cytotoxicity using the MTT assay. RAW 264.7 cells. After 24 h of exposure, CEA showed a non-cytotoxic effect at 400 µg/mL. To evaluate its anti-inflammatory potential, CEA was assessed for its capacity to inhibit nitric oxide (NO) production in LPS-stimulated RAW 264.7 macrophage cells (Table 5). LPS (1 μg/mL) significantly increased nitrite levels, reflecting elevated NO production. CEA treatment produced a concentration-dependent reduction in nitrite accumulation (*p* < 0.05), with an IC_50_ of 62.68 µg/mL (Table 5), demonstrating effective inhibition of LPS-induced NO production. However, it showed lower activity than indomethacin.

### 3.10. Formulation and Stability Evaluation

The CEA was selected for formulation due to its chemical profile and in vitro biological activities. Increasing the extract concentration led to a more yellow appearance, lower pH, and reduced viscosity of the lotion. These changes were attributed to the extract’s natural yellow-brown color and its content of phenolic and organic acids, such as protocatechuic, salicylic, citric, tartaric, and malic acids [5]. To maintain a skin-compatible pH (5–6), increased amounts of triethanolamine (TEA) were added. Despite pH adjustment, viscosity decreased with higher extract levels, likely due to the presence of excess protons and electrolytes interfering with the ionization and expansion of Carbopol polymer chains, which are sensitive to both pH and ionic strength [39,40,41].

In the preliminary stability test, all formulations showed no phase separation after centrifugation at 3000 rpm for 30 min. They were then subjected to an accelerated stability test using six heating–cooling cycles. Color, pH, and viscosity were evaluated at the initial point and after one, three, and six cycles (Table 6). After six cycles, the color of all formulations remained unchanged. F1 showed a slight increase in pH, while F2, F3, and F4 exhibited minor decreases, though none were statistically significant. Viscosity decreased in all formulations over time, but no phase separation was observed. Despite slight changes in pH and viscosity, all formulations remained within acceptable limits and were considered stable.

### 3.11. Clinical Evaluation

#### 3.11.1. Skin Irritation Test

The safety assessment was conducted using a single application closed patch test on 30 volunteers. After patch removal, volunteers showed slight irritation by SLS, with an M.I.I. of 0.33. Only one volunteer had a mild reaction to the 1.0% *w*/*w* CEA lotion. The patch test results confirmed the safety of CEA-containing formulations, with an M.I.I. value of 0.03 for 1.0% CEA lotion, indicating non-irritation. This finding supports the use of cocoa pod husk extract in cosmetic formulations. The safety may be attributed to the natural composition of the extract, which contains non-toxic phenolics and flavonoids such as ferulic acid, protocatechuic acid, and kaempferol, known for their anti-inflammatory and skin-soothing properties [5,29]. Thus, both the base lotion and formulations containing up to 1.0% *w*/*w* extract were classified as non-irritating. For the clinical evaluation of moisturizing, transepidermal water loss, melanin index, and skin surface analysis, including skin smoothness (SEsm), skin roughness (SEr), scaliness (SEsc), and wrinkle (SEw), 29 volunteers who showed no sign of skin irritation were enrolled. One participant withdrew, leaving twenty-eight who completed the study. Each volunteer’s left inner forearm was divided into four 3 × 3 cm areas for application of base lotion (F1) and lotions with 0.01% (F2), 0.1% (F3), and 1.0% (F4) cocoa pod husk extract, applied twice daily for 4 weeks. Non-invasive instruments were used to assess skin condition at baseline (T0) and after 1 (T1), 2 (T2), 3 (T3), and 4 (T4) weeks. No adverse reactions were observed, confirming the formulations’ safety.

#### 3.11.2. Skin Hydration

**Figure 4:**
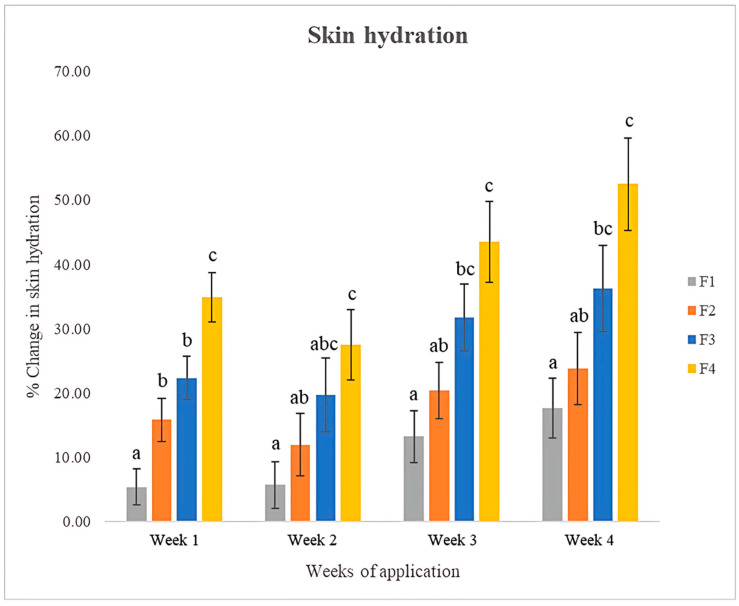
Percent changes in skin hydration after the application of lotions for 1, 2, 3, and 4 weeks. Different letters in the same weeks of application indicate significant differences (*p* < 0.05). Values represent mean ± SEM.

Skin hydration was measured using a Corneometer^®^ CM 825. As shown in Figure 4, lotions containing CEA significantly increased skin hydration compared to the base lotion from the first week (*p* < 0.05). Notably, F4 increased hydration by 34.83% after one week, significantly more than the other formulations (*p* < 0.05). By the end of the study, F4 showed a 52.48% improvement, significantly higher than F1 (*p* < 0.001) and F2 (*p* = 0.001). All extract-containing formulations enhanced skin moisture compared to baseline, indicating the extract’s moisturizing potential. The dose-dependent increase in skin hydration, especially with F4 (1.0% CEA), may be linked to the hydroxyl-rich polyphenols identified in Table 3. Compounds such as ferulic acid, chlorogenic acid, and procyanidins possess multiple hydroxyl groups capable of forming hydrogen bonds with water molecules, enhancing moisture retention in the stratum corneum [42,43]. Moreover, apigenin-7-O-glucoside and kaempferol, known flavonoids in CEA, have been reported to stimulate aquaporin expression and enhance skin barrier hydration, contributing to the sustained increase in water content over 4 weeks. By the way, the base lotion’s hydrating effect could be due to the presence of humectants such as glycerin and propylene glycol.

#### 3.11.3. Transepidermal Water Loss (TEWL)

**Figure 5:**
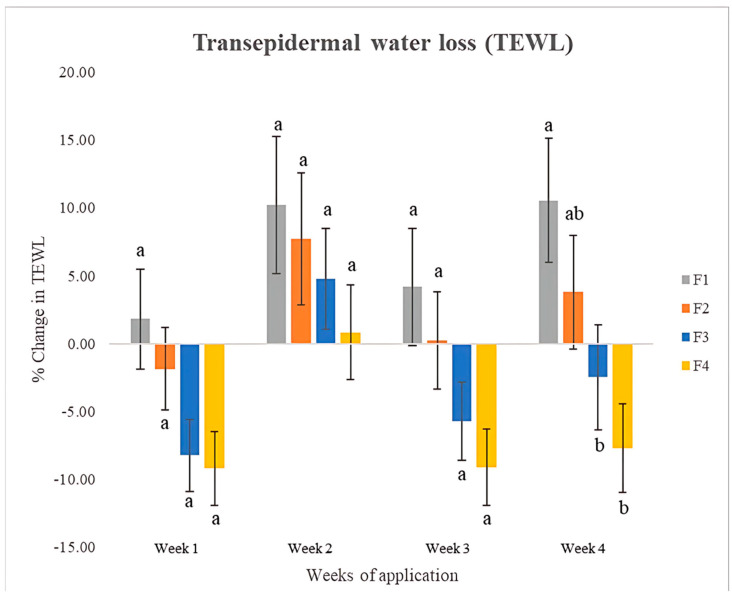
Percent changes in transepidermal water loss (TEWL) after the application of lotions for 1, 2, 3, and 4 weeks. Different letters in the same weeks of application indicate significant differences (*p* < 0.05). Values represent mean ± SEM.

Transepidermal water loss (TEWL) was measured using a Tewameter^®^ TM 300. Among the formulations containing CEA, F4 showed the greatest reduction in TEWL, although the difference was not statistically significant. However, after four weeks of application, both F3 (−2.49%) and F4 (−7.73%) significantly reduced TEWL compared to the base lotion (*p* < 0.05), as shown in Figure 5. The reduction in TEWL observed with F3 and F4 may be directly associated with the barrier-enhancing effects of polyphenols in the extract. Ferulic acid and procyanidins have been shown to strengthen the lipid matrix of the skin barrier by upregulating tight junction proteins and promoting ceramide synthesis [44,45]. The presence of resveratrol also plays a role, as it has been demonstrated to enhance epidermal barrier repair and reduce inflammation, indirectly minimizing water loss [46]. The improved skin barrier integrity reflected by reduced TEWL aligns with these compound mechanisms [46,47].

#### 3.11.4. Skin Pigmentation

**Figure 6:**
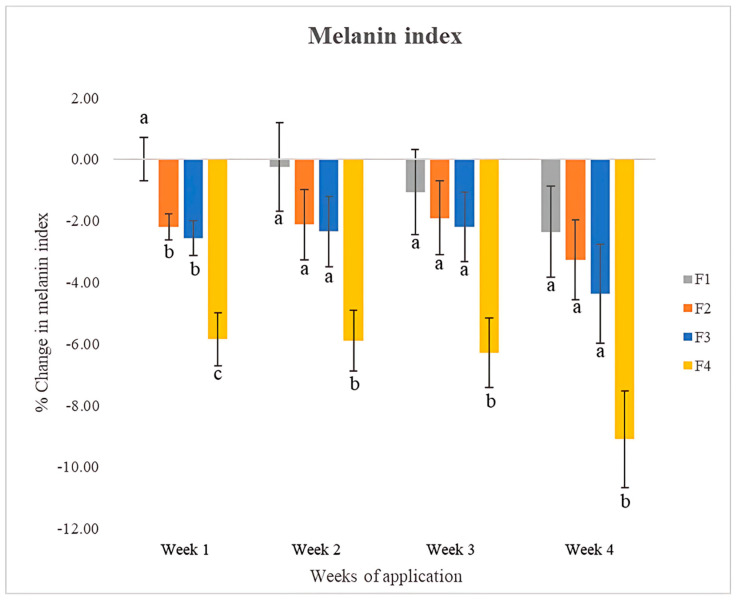
Percent changes in melanin index after the application of lotions for 1, 2, 3, and 4 weeks. Different letters in the same weeks of application indicate significant differences (*p* < 0.05). Values represent mean ± SEM.

After one week of application, lotions containing CEA showed a significantly greater reduction in melanin index compared to the base lotion (*p* < 0.05), as shown in Figure 6. By the end of the study, F4 resulted in a 9.10% decrease in melanin index, significantly more than other formulations (*p* < 0.05), and consistently exhibited the strongest skin-lightening effect throughout the study. The reduction in melanin index from baseline indicated visible skin brightening, with higher extract concentrations yielding greater effects. The observed decrease in melanin index, especially in F4, is consistent with the in vitro tyrosinase inhibition and is likely due to the synergistic activity of several bioactive flavonoids in CEA. Specifically, naringenin, kaempferol, apigenin-7-O-glucoside, and procyanidins B1 and B2 are known to inhibit melanin synthesis through tyrosinase inhibition, melanogenesis suppression, and modulation of MITF (microphthalmia-associated transcription factor) expression [36,44,48,49]. Additionally, resveratrol acts as a competitive inhibitor of tyrosinase and disrupts melanosome transfer, contributing to the skin-lightening effects seen in the clinical trial [46,47].

#### 3.11.5. Skin Surface Analysis

**Figure 7:**
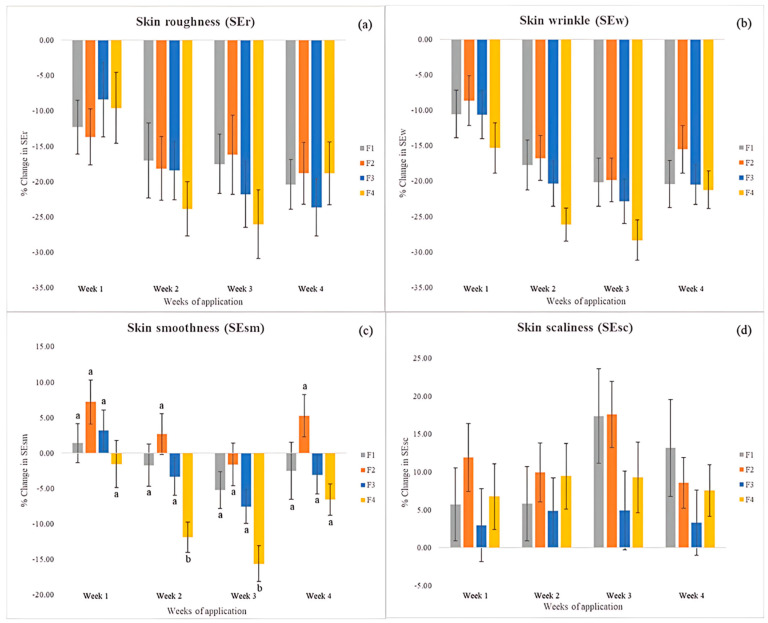
Percent changes in skin roughness (**a**), skin wrinkle (**b**), skin smoothness (**c**), and skin scaliness (**d**) after the application of lotions for 1, 2, 3, and 4 weeks. Different letters in the same weeks of application indicate significant differences (*p* < 0.05). Values represent mean ± SEM.

Skin surface analysis was conducted using the Visioscan^®^ VC98, evaluating the Surface Evaluation of Living Skin (SELS) parameters: roughness (SEr), wrinkles (SEw), smoothness (SEsm), and scaliness (SEsc). No significant differences in skin roughness reduction were observed among formulations (Figure 7a). However, all formulations showed a significant reduction in roughness compared to baseline from the first week of application (*p* < 0.05), as shown in Table 7.

Skin wrinkles significantly decreased after one week of lotion application across all formulations (*p* < 0.01; Figure 7b, Table 8). F4 exhibited the greatest wrinkle reduction, although not significantly different from the others.

The lower skin smoothness values indicate smoother skin. After one week, only F4 showed a reduction in smoothness values, though not statistically significant (Figure 7c). By week 2, F4 significantly improved skin smoothness compared to baseline (*p* < 0.001; Table 9) and demonstrated the highest improvement throughout the study, but there was no significant difference.

**Figure 8:**
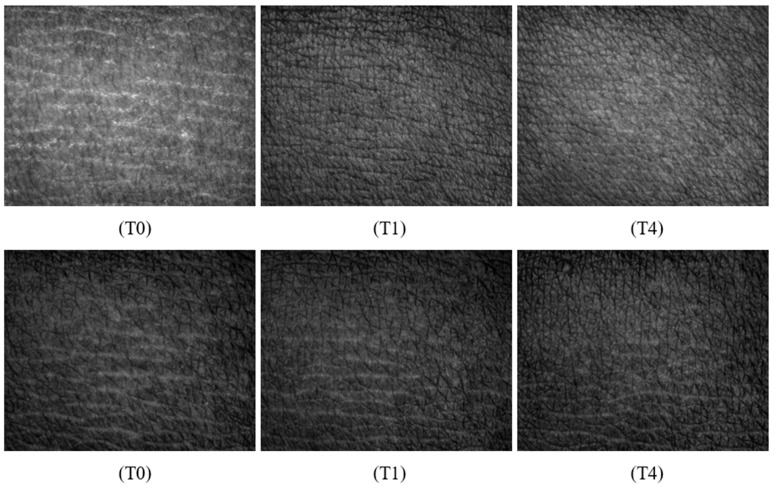
Two representative volunteers’ skin at the baseline (T0), after 1 week (T1), and after 4 weeks (T4) of application of lotion containing 1.0% *w*/*w* of CEAt (F4).

At the end of the study, no significant differences in skin scaliness were observed among formulations (Figure 7d). However, F4 showed a noticeable improvement in scaliness compared to baseline (Figure 8).

Skin aging is influenced by intrinsic factors (genetics and hormones) and extrinsic factors such as UV exposure, air pollution, smoking, and poor nutrition. It is characterized by skin dryness, wrinkles, hyperpigmentation, and rough texture [50,51]. The improvement in roughness, wrinkles, and smoothness, particularly in F4, can be linked to the antioxidant and anti-collagenase activities of the extract. Compounds such as procyanidin C1, ferulic acid, and kaempferol have strong antioxidant properties that reduce oxidative stress, a key driver of collagen degradation and wrinkle formation [52,53,54,55]. Furthermore, the anti-collagenase effect observed in vitro may result from direct enzyme inhibition by flavonoids and phenolic acids, which protect the extracellular matrix by chelating metal ions in collagenase’s active site [32,38]. These actions help maintain collagen structure and elasticity, reflected in reduced SEw and improved SEsm values. The observed skin texture improvements correlate well with the known biological activities of these compounds. Although the current study demonstrates promising short-term effects, extended clinical trials (≥8 weeks) are essential to fully substantiate the extract’s anti-aging efficacy, as supported by previous findings [52,56].

## 4. Conclusions

This study demonstrates that cocoa pod husk extract prepared using acidified ethanol (CEA) possesses significant potential as a multifunctional cosmetic active ingredient. The extract exhibited strong antioxidant, anti-collagenase, and tyrosinase inhibitory activities in vitro, which are attributable to its rich composition of polyphenols and flavonoids, including ferulic acid, kaempferol, and procyanidins. Clinical evaluations further confirmed the extract’s efficacy in improving skin hydration, reducing transepidermal water loss, lightening pigmentation, and enhancing skin surface parameters such as roughness, wrinkle depth, and smoothness. While the formulation containing 1.0% CEA (F4) consistently demonstrated the most pronounced effects, the improvements observed over the 4-week period suggest a dose-dependent response rather than statistical superiority. Given that more substantial anti-wrinkle effects have been reported in longer trials (8–12 weeks), future studies with extended treatment durations are recommended to validate and expand upon these findings. Overall, cocoa pod husk extract represents a promising natural candidate for incorporation into skin care formulations aimed at hydration, brightening, and anti-aging.

## Figures and Tables

**Table 1:** Formula of base lotion (F1) and lotion containing cocoa pod husk extract (F2, F3, F4).

Phase	Ingredient	INCI Name	Amount (% *w*/*w*)				Function
			F1	F2	F3	F4	
A	Deionized Water	Water	81.7	81.64	81.5	80.3	Solvent
	Disodium EDTA	Disodium EDTA	0.1	0.1	0.1	0.1	Chelating Agent
	Carbopol Ultrez 21	Acrylates/C10-30 Alkyl Acrylate Crosspolymer	0.5	0.5	0.5	0.5	Thickening Agent
	Glycerin	Glycerin	1.0	1.0	1.0	1.0	Humectant
B	PEG-40 Hydrogenated Castor Oil	PEG-40 Hydrogenated Castor Oil	2.5	2.5	2.5	2.5	Emulsifier
	Dimethicone	Dimethicone	1.0	1.0	1.0	1.0	Sensory Modifier
	Isononyl Isononanoate	Isononyl Isononanoate	4.0	4.0	4.0	4.0	Emollient
	Caprylic/Capric Triglyceride	Caprylic/Capric Triglyceride	4.0	4.0	4.0	4.0	Emollient
	Silk Lotion Maker	Bis-PEG/PPG-16/16 PEG/PPG16/16 Dimethicone	1.0	1.0	1.0	1.0	Sensory Modifier, Co-emulsifier
C	Propylene Glycol	Propylene Glycol	3.0	3.0	3.0	3.0	Solvent, Humectant
	Cocoa Pod Husk Extract	Theobroma Cacao Husk Extract	-	0.01	0.1	1.0	Active
D	Phenoxyethanol (and) Ethyhexylglycerin	Phenoxyethanol (and) Ethyhexylglycerin	1.0	1.0	1.0	1.0	Preservative
E	Triethanolamine	Triethanolamine	0.2	0.25	0.3	0.6	pH Adjuster

**Table 2:** Total phenolic content (TPC) and total flavonoid content (TFC) of CPH extracts by different solvents.

Samples	TPC (mg GAE/g Extract)	TFC (mg QE/g Extract)
CE	157.491 ± 7.941 ^a^	2.22 ± 0.20 ^a^
CEA	170.977 ± 7.412 ^b^	3.91 ± 0.27 ^b^

^a,b^ Different letters in the same column indicate significant differences (*p* < 0.05).

**Table 4:** Antioxidant, anti-glycation, anti-tyrosinase, and anti-collagenase activities of cocoa pod husk extracted by different extraction solvents.

Sample	DPPH Assay (IC_50_, μg/mL)	FRAP Assay (mg AAE/g Extract)	Anti-Glycation Activity (IC_50_, µg/mL)	Tyrosinase Inhibitory Activity (IC_50_, mg/mL)	Anti-Collagenase Activity (IC_50_, mg/mL)
CE	6.91 ± 0.14 ^a^	173.44 ± 4.06 ^b^	ND	ND	ND
CEA	5.83 ± 0.11 ^b^	234.17 ± 4.01 ^a^	66.96 ± 0.57 ^a^	9.51 ± 0.01 ^a^	0.43 ± 0.01 ^a^
Ascorbic acid	1.48 ± 0.06 ^c^	ND	ND	ND	ND
Quercetin	ND	ND	3.87 ± 0.19 ^b^	ND	ND
Kojic acid	ND	ND	ND	0.01 ± 0.00 ^b^	ND
EGCG	ND	ND	ND	ND	0.07 ± 0.01 ^b^

Values are given as mean ± SD from triplicate. ND means not determined. Different letters in the same column indicate significant differences (*p* < 0.05).

**Table 5:** Nitric oxide (NO) production inhibition of CEA.

Sample	Concentration (µg/mL)	% of NO Inhibition	Sample (IC_50_, µg/mL)
CEA	6.25	20.42 ± 1.34	62.68
	12.5	31.55 ± 3.41	
	25	39.47 ± 2.78	
	50	51.43 ± 3.81	
	100	88.41 ± 0.05	
	200	98.28 ± 1.03	
Indomethacin			13.23

**Table 6:** Stability testing results of F1, F2, F3, and F4 lotions from six cycles of heating–cooling.

Formulation	Parameter	T0	T1	T3	T6
F1	Color	White	White	White	White
	pH	5.26 ± 0.01	5.28 ± 0.01	5.29 ± 0.01	5.29 ± 0.01
	Viscosity (cP)	6564.33 ± 88.95	6521.33 ± 37.61	6512.00 ± 25.06	6507.33 ± 21.50
F2	Color	White	White	White	White
	pH	5.30 ± 0.01	5.28 ± 0.01	5.27 ± 0.01	5.25 ± 0.00
	Viscosity (cP)	6530.67 ± 51.79	6452.67 ± 25.11	6378.33 ± 19.09 *	6321.33 ± 28.29 *
F3	Color	Ivory	Ivory	Ivory	Ivory
	pH	5.30 ± 0.01	5.28 ± 0.01	5.27 ± 0.01	5.24 ± 0.01
	Viscosity (cP)	6450.00 ± 43.00	6373.67 ± 41.59 *	6276.00 ± 35.76 *	6131.33 ± 50.16 *
F4	Color	Cream	Cream	Cream	Cream
	pH	5.33 ± 0.01	5.30 ± 0.01	5.26 ± 0.01	5.24 ± 0.01
	Viscosity (cP)	6285.05 ± 22.00	6252.00 ± 45.20	6012.00 ± 15.04 *	5976.31 ± 25.23 *

Spindle no.6, 140 rpm, 25 °C, %Torque > 80%. * Indicates significant differences (*p* < 0.05), compared with values at T0.

**Table 7:** Skin roughness (SEr) values at the baseline, after the application of lotions for 1, 2, 3, and 4 weeks.

Formulation	Skin Roughness (SEr) Value				
	T0	T1	T2	T3	T4
F1	2.48 ± 0.09	2.15 ± 0.10 *	2.03 ± 0.13 *	2.00 ± 0.09 *	1.96 ± 0.10 *
F2	2.71 ± 0.12	2.27 ± 0.10 *	2.16 ± 0.12 *	2.22 ± 0.15 *	2.22 ± 0.18 *
F3	2.41 ± 0.11	2.12 ± 0.09 *	1.94 ± 0.11 *	1.85 ± 0.12 *	1.85 ± 0.14 *
F4	2.04 ± 0.08	1.81 ± 0.10 *	1.55 ± 0.09 *	1.50 ± 0.10 *	1.65 ± 0.11 *

At the baseline (T0), after 1 week (T1), after 2 weeks (T2), after 3 weeks (T3), after 4 weeks (T4). Values are given as mean ± SEM. * Indicates significant differences (*p* < 0.05), compared with values at T0.

**Table 8:** Skin wrinkle (SEw) values at the baseline, after the application of lotions for 1, 2, 3, and 4 weeks.

Formulation	Skin Wrinkle (SEw) Value				
	T0	T1	T2	T3	T4
F1	62.79 ± 3.40	54.03 ± 1.84 *	49.69 ± 2.17 *	48.31 ± 2.16 *	48.07 ± 2.06 *
F2	59.38 ± 2.97	52.80 ± 2.44 *	47.95 ± 2.05 *	46.18 ± 2.14 *	49.02 ± 2.46 *
F3	58.05 ± 2.42	51.11 ± 2.39 *	45.46 ± 2.14 *	44.04 ± 2.20 *	45.68 ± 2.15 *
F4	59.16 ± 2.31	49.00 ± 2.09 *	43.20 ± 1.98 *	41.83 ± 2.12 *	46.38 ± 2.35 *

At the baseline (T0), after 1 week (T1), after 2 weeks (T2), after 3 weeks (T3), after 4 weeks (T4). Values are given as mean ± SEM. * Indicates significant differences (*p* < 0.05), compared with values at T0.

**Table 9:** Skin smoothness (SEsm) values at the baseline, after the application of lotions for 1, 2, 3, and 4 weeks.

Formulation	Skin Smoothness (SEsm) Value				
	T0	T1	T2	T3	T4
F1	147.26 ± 6.89	145.61 ± 4.46	141.77 ± 5.57	137.00 ± 5.43 *	140.67 ± 6.98
F2	156.28 ± 6.95	165.58 ± 7.58	157.71 ± 6.44	150.26 ± 5.25	162.18 ± 6.96
F3	170.35 ± 6.78	174.73 ± 7.89	163.02 ± 6.80	155.92 ± 6.38 *	163.46 ± 6.47
F4	185.54 ± 7.23	179.95 ± 7.29	162.36 ± 7.18 *	155.17 ± 6.92 *	172.40 ± 7.33 *

At the baseline (T0), after 1 week (T1), after 2 weeks (T2), after 3 weeks (T3), after 4 weeks (T4). Values are given as mean ± SEM. * Indicates significant differences (*p* < 0.05), compared with values at T0.

## Data Availability

The original contributions presented in this study are included in the article. Further inquiries can be directed at the corresponding author.
